# Eating behaviors in preschoolers with autism spectrum disorders: associations with developmental and behavioral features and parental mental health

**DOI:** 10.3389/fpsyt.2026.1902816

**Published:** 2026-10-15

**Authors:** Esra Hoşoğlu, Ayhan Bilgiç, Ömer Faruk Akça, Mahmut Sami Tuzcu

**Affiliations:** 1 Department of Child and Adolescent Psychiatry, Meram Faculty of Medicine, Necmettin Erbakan University, Konya, Türkiye; 2 36th Family Health Center of Karatay, Konya, Türkiye

**Keywords:** autism, autism spectrum disorder, eating behaviors, eating problems, maternal mental health, parental mental health, preschool period

## Abstract

**Introduction:**

Eating problems are highly prevalent in children with autism spectrum disorder (ASD). However, their profile during the preschool period—a critical window for establishing lifelong eating habits—remains poorly understood. This study aimed to compare the eating behaviors of preschool children with ASD and typically developing (TD) peers and to identify associated developmental, behavioral, and parental factors during this formative stage.

**Methods:**

This case-control study included 50 preschool children with ASD (aged 2–6 years) recruited from a child and adolescent psychiatry outpatient clinic and 43 age-matched TD children recruited from family health centers. ASD diagnoses were confirmed by child and adolescent psychiatrists according to DSM-5 criteria. Parents completed the Children’s Eating Behavior Questionnaire (CEBQ), the Aberrant Behavior Checklist (ABC), and the Brief Symptom Inventory (BSI). Child assessments included the Childhood Autism Rating Scale (CARS) and the Ankara Developmental Screening Inventory (ADSI). Group differences were examined using univariate analyses and multivariate analyses of covariance (MANCOVA). Within the ASD group, correlations and multivariable linear regressions examined associated factors. Complete-case sensitivity analyses were performed for variables with missing data.

**Results:**

No significant between-group differences were observed across CEBQ subscales after adjustment for baseline differences (all p > 0.05). Within the ASD group, hyperactivity/noncompliance was positively correlated with satiety responsiveness in the primary analysis (r_s_ =0.449, FDR-adjusted p=0.022), but not after FDR correction in the complete-case analysis (r_s_ =0.431, adjusted p=0.066). Fine motor and cognitive-language developmental levels showed nominal associations with specific eating behavior dimensions in multivariable regressions (β=−0.333 to 0.404, p=0.012–0.043), but none remained significant after FDR correction. Complete-case analyses yielded generally similar effect estimates, with no regression associations surviving FDR correction.

**Conclusion:**

Parent-reported CEBQ dimensions did not differ significantly between preschool children with ASD and TD peers. Within the ASD group, developmental and behavioral characteristics showed potential associations with specific eating behaviors; however, these findings did not consistently withstand multiple-testing correction. Given the cross-sectional design and relatively small sample, the findings should be interpreted cautiously and require replication in larger longitudinal studies.

## 1 Introduction

Autism Spectrum Disorder (ASD) is a neurodevelopmental disorder characterized by impairments in social interaction and communication, as well as the presence of restricted and repetitive behaviors and interests (1). In addition to these core diagnostic features, comorbid psychiatric and medical conditions are frequently observed in children with ASD, with eating and feeding problems being among the most common (2). Although eating and feeding difficulties are highly prevalent in ASD, their nature and determinants in early childhood remain poorly understood, highlighting a critical need for focused research.

The preschool period is recognized as a critical window for establishing eating behaviors and dietary patterns (3–5). These behaviors tend to persist from childhood through adolescence and into adulthood (4, 6). Given this persistence, problematic eating behaviors at this early stage can lead to long-term health and weight-related issues (7, 8). Eating behaviors are commonly categorized as either food approach or food avoidant. Approach behaviors, such as food responsiveness and emotional overeating, are linked to a greater risk of overweight and obesity. In contrast, avoidant behaviors like picky eating and high satiety responsiveness are associated with underweight and inadequate nutritional intake during the preschool years (4, 8). Beyond physical health, problematic eating generates significant stress for parents and can negatively affect family relationships (4, 9).

Atypical eating behaviors in children with ASD have been recognized since Kanner’s first clinical descriptions (10). Subsequent research has consistently reported that eating and feeding problems are common in this population, with prevalence rates ranging from 46% to 89% (11–14). More recently, a large study involving 1,462 children with ASD reported atypical eating behaviors in 70.4% of participants, while a recent systematic review estimated the prevalence of food selectivity at approximately 63% among children with ASD (15, 16). However, despite growing attention to this issue, research focusing specifically on the early developmental and preschool periods remains limited and yields inconsistent findings. Some studies have identified higher rates of food selectivity, refusal, and mealtime difficulties in preschoolers with ASD (14, 17–19), while others have reported only partial or non-significant group differences (20–23). These inconsistencies underscore the need for further research focusing specifically on eating behaviors during the preschool years, a critical period when lifelong eating habits and family mealtime routines are established.

These mixed findings highlight the complex and multifactorial nature of eating problems in ASD, reflecting both individual and environmental influences. Nevertheless, the underlying causes of these difficulties remain poorly understood. Previous research suggests that these difficulties may result from a complex interplay of core autistic traits, sensory sensitivities, comorbid medical conditions, and environmental factors (24, 25). In TD children, eating behaviors are largely shaped through social learning and modeling of others. However, children with ASD may struggle with this process due to deficits in social communication and imitation skills (25). Consequently, they may have difficulty expressing their food preferences and needs or understanding parental mealtime expectations. Sensory sensitivities, such as heightened responses to texture, taste, or smell, can contribute to food refusal, while insistence on sameness and adherence to routines may further exacerbate mealtime challenges (24, 26, 27). Moreover, developmental delays and behavioral difficulties have also been associated with increased eating problems in children with ASD (26, 28).

Parents play a critical role in shaping children’s eating behaviors. Mealtimes are fundamentally social experiences, and children’s food preferences are significantly influenced by observing and modeling the eating behaviors and food intake patterns of parents, peers, and siblings (3, 29). For instance, Birch (30) demonstrated that preschoolers increased their intake of previously disliked vegetables after observing their peers eating them. Parental feeding practices are another key influence. Carper et al. (31) found that in young girls, the perception of parental pressure to eat was associated with greater dietary restriction and emotional disinhibition. Furthermore, parental mental health is a significant factor that can shape these feeding practices. Research has shown correlations between maternal anxiety and the use of restrictive feeding practices (32), as well as between maternal depression and increased pressure to eat (33). Although limited, recent studies suggest a similar link in families of children with ASD, where higher levels of parental anxiety and depression are associated with more problematic child feeding behaviors and the use of controlling strategies (34, 35). Just as parental psychopathology may influence children’s eating behaviors, feeding problems can also lead parents to experience guilt and increased levels of stress (36). However, more research is needed to better understand these associations and their implications for intervention.

The preschool period represents a formative stage for the development of eating behaviors, and eating problems emerging during this period may persist and become increasingly resistant to change if not properly addressed (3, 37). This persistence underscores the need for early identification and comprehensive evaluation of eating difficulties in this age group to prevent long-term nutritional, behavioral, and psychosocial consequences (37). Although eating and feeding difficulties in ASD have been widely documented, much less is known about their characteristics and associated factors during the preschool years, a critical developmental phase for establishing lifelong eating habits and family mealtime routines. Moreover, parent-related factors, particularly parental mental health, have not been sufficiently investigated in relation to children’s eating behaviors in ASD. Accordingly, we hypothesized that (1) eating behaviors would differ between preschool children with ASD and their typically developing peers, and (2) within the ASD group, these difficulties would be associated with the autism symptom severity, age, child’s developmental level, behavioral characteristics, and parental mental health.

## 2 Materials and methods

### 2.1 Participant and procedures

The patient group consisted of 50 children with ASD aged 2 to 6 years who applied to a child and adolescent mental health service in Türkiye. Clinical diagnoses of ASD were made according to the DSM- 5 criteria by experienced child and adolescent psychiatrists. The control group was composed of 43 healthy children without ASD who were consecutively recruited from a primary care health center in Türkiye. As no *a priori* sample size calculation was performed, a *post hoc* sensitivity analysis was conducted to determine the minimum detectable effect size given the achieved sample size (50 children with ASD and 43 typically developing peers). The analysis indicated that, assuming a two-sided α of 0.05 and 80% power, the minimum detectable between-group effect size was Cohen’s d = 0.59. Accordingly, smaller but potentially clinically meaningful effects may have remained undetected. Families were informed about the study, and children whose parents provided written informed consent and met the inclusion and exclusion criteria were enrolled. To exclude ASD, intellectual disability and communication disorders, each child in the control group was evaluated by a clinician. We excluded children who were taking appetite-affecting drugs, had chronic diseases (e.g. diabetes, cerebral palsy, epilepsy, GI diseases, etc.) and whose parents were currently receiving psychiatric treatment in both groups, because ongoing treatment may reduce or mask current psychiatric symptoms, potentially confounding the association between parental psychopathology (assessed using the BSI) and child eating behaviors. In the control group, children whose mother, father, or siblings had ASD were also excluded.

The severity of ASD was assessed using the Childhood Autism Rating Scale (CARS) in the ASD group only. Developmental level was assessed in both groups using the Ankara Developmental Screening Inventory (ADSI). In both groups, mothers and fathers completed the Children’s Eating Behavior Questionnaire (CEBQ) and the Aberrant Behavior Checklist (ABC) for their children, as well as the Brief Symptom Inventory (BSI) for themselves. The physician responsible for recruiting the control participants was not blinded to the study objectives and served as a co-investigator. During recruitment, the physician was responsible only for determining eligibility according to the predefined inclusion and exclusion criteria, whereas eating behaviors were assessed using parent-completed questionnaires (CEBQ), independent of the recruiting physician’s involvement.

All procedures performed in studies involving human participants were in accordance with the ethical standards of the institutional and/or national research committee and with the 1964 Helsinki Declaration and its later amendments or comparable ethical standards. This study was approved by the Ethics Committee of Necmettin Erbakan University, Meram Faculty of Medicine (Approval No: 2017/902; Date: 28.04.2017). The parents who agreed to have their children take part were provided their written informed consent.

### 2.2 Scales and measures

#### 2.2.1 Socio-demographic data form

The form developed by the researchers to collect data about socio-demographic characteristics of participants such as the age, gender, number of siblings, family structure, parent’s educational levels, parent’s ages, economic status.

#### 2.2.2 Children eating behavior questionnaire

The CEBQ is a 35-item parent-report measure developed by Wardle et al. (2001), with items rated on a 5-point Likert scale (38). There are sub-dimensions of food responsiveness, emotional overeating, enjoyment of food, desire to drink, satiety responsiveness, slowness in eating, emotional undereating and food fussiness. The reliability and validity study of the CEBQ for Turkish children was carried out (39).

#### 2.2.3 Childhood autism rating scale

This scale has been developed by Schopler et al. (1980) to differentiate autistic children from children with other developmental disorders (40). The Turkish validity and reliability studies were conducted by Sucuoğlu et al. (1996) and İncekaş Gassaloğlu et al.(2016) (41, 42). The scale consists of 15 items, each scored on a 1 to 4 scale in 0.5-point increments (i.e., 1, 1.5, 2, 2.5, 3, 3.5, and 4). The evaluation can be done through observation, parent interviews and review of records. A total score of 30 and above is considered clinically meaningful for autism.

#### 2.2.4 The aberrant behavior checklist

The scale was developed by Aman et al. (1985) to assess the problematic behaviors and the effects of the treatments on these problematic behaviors observed in individuals with intellectual disabilities (43). Karabekiroglu and Aman conducted the Turkish reliability and validity study for children aged 1–4 years (44). The scale consists of 58 items and each item is scored between 0 and 3. There are five sub-scales: hyperactivity/noncompliance, irritability/agitation/crying, lethargy/social withdrawal, stereotypic behaviors and inappropriate speech.

#### 2.2.5 Ankara developmental screening inventory

The ADSI is an inventory developed to assess the developmental levels of Turkish children aged 0–6, based on caregiver report. It consists of 154 items answered as yes/no/do not know. Four areas, namely Language-Cognitive, Fine-Motor, Gross-Motor and Social Skill-Self Care are evaluated, and total development score is obtained. The ADSI provides a general developmental score, reflecting the child’s overall developmental level, which was primarily used in this study to assess participants’ developmental status at the time of evaluation (45).

#### 2.2.6 Brief symptom inventory

The BSI is a 53-item multidimensional symptom screening scale designed by Derogatis (46). Each item of the scale is rated between 0 and 4 points. The Turkish adaptation study of the scale revealed five distinct factors (anxiety, depression, negative self, somatization and hostility) (47). Higher scores are associated with higher possibility of a psychopathology.

### 2.3 Statistical analysis

All statistical analyses were performed using IBM SPSS Statistics version 20.0 (IBM Corp., Chicago, IL, USA). Prior to conducting the statistical analyses, missing values were imputed using the series mean method, whereby each missing value was replaced with the mean of the corresponding variable. To assess the potential influence of this imputation approach, analyses involving variables with missing data were repeated using complete cases without imputation. For the evaluation of the ADSI data, a ratio was calculated by dividing the developmental age obtained from the ADSI by the chronological age of each participant. This ratio corresponds to the Developmental Quotient (DQ), a widely used index in early childhood developmental assessment. A value of 1.0 (or 100 when multiplied by 100) indicates age-appropriate development, whereas values ​​below 1.0 indicate a developmental delay relative to chronological age. Current developmental tests are increasingly based on age-standardized normative scores. However, given the lack of a suitable tool in Türkiye at the time of the study to identify developmental gaps in the preschool period, the use of the ADSI developmental age ratio was considered the most appropriate option. Although this approach is not the most ideal approach, it is frequently used in studies conducted in our country and published in the international literature (e.g., Bilgiç et al. (48)).

The normality of data distribution was assessed using the Kolmogorov–Smirnov test. Group comparisons were performed using independent sample t-tests and Mann–Whitney U tests for continuous variables, and chi-square tests for categorical variables. Continuous variables that were not normally distributed were natural log transformed before multivariate analysis of covariance (MANCOVA) and multivariable linear regression analyses. Group differences in eating behaviors were further examined using MANCOVA. In the first model, sociodemographic characteristics that differed significantly between the ASD and TD groups were included as covariates. In a subsequent model, developmental level was additionally included as a covariate to examine whether the group effect on eating behaviors changed after accounting for developmental differences between the groups. To minimize multicollinearity among developmental measures, the overall ADSI developmental quotient was used to represent developmental level rather than the individual ADSI subscale scores.

Correlation and multivariable linear regression analyses were performed in the ASD group. Pearson correlation coefficients were calculated for normally distributed variables, whereas Spearman’s rank correlation coefficients were used for non-normally distributed variables, based on the results of the Kolmogorov–Smirnov test. To account for multiple testing, the Benjamini–Hochberg false discovery rate (FDR) procedure was applied separately for each CEBQ subscale. Specifically, a separate correction was performed for the set of correlation coefficients calculated between each CEBQ subscale and the corresponding clinical, developmental, and parental variables.

Separate multivariable linear regression analyses were performed for each CEBQ subscale. For the regression analyses conducted within the ASD group, variables associated with each CEBQ dimension at p<.20 in univariate analyses were considered as candidate predictors. Given the ASD sample size (n=50), model complexity was restricted to a maximum of five predictors to reduce the risk of overfitting. When more than five variables met the screening criterion, predictors were selected based on their theoretical relevance to the specific eating behavior dimension, while avoiding the simultaneous inclusion of conceptually overlapping variables. When five or fewer candidate predictors were identified, all were retained in the corresponding model. To address multiplicity across the regression analyses, the Benjamini–Hochberg false discovery rate (FDR) procedure was applied jointly to the p values of all predictor coefficients tested across the eight multivariable regression models. A two-tailed p-value of <0.05 was considered statistically significant.

## 3 Results

The study consisted of 93 children, including 50 children with ASD and 43 TD children. The mean age was namely 37.40 ± 9.88 months for patient group and 34.3 ± 8.05 months for control and there was no significant difference in mean age between the groups (p*=*0.112). There was a predominance of males in both groups (ASD group: 41 boys/50 total; Control group: 33 boys/43 total). Parental education status and levels of income were similar. The fathers and mothers were significantly younger in the control group than in the ASD group. **Table 1** demonstrates the demographic characteristics of the two groups.

**Table 1:** Sociodemographic features.

Characteristic	ASD (*n* = 50)	TD (*n* = 43)	*t/z^2^*	Effect size	*P*
Sex (male/female)	41/9	33/10		φ = 0.07	0.531
Age	37.40 ± 9.88	34.3 ± 8.05	1.603^a^	d = 0.33	0.112
Mother’s age	32.88 ± 5.23	29.70 ± 4.38	3.149^a^	d = 0.66	0.002
Father’s age	36.46 ± 6.43	32.11 ± 4.46	-3.372^b^	r = -0.35	0.001
Mother’s education level (year)	10.17 ± 4.63	9.45 ± 4.64	-0.748^b^	r = -0.08	0.454
Father’s education level (year)	10.50 ± 4.26	9.55 ± 4.14	-1.005^b^	r = -0.11	0.315
Income	2.46 ± 0.76	2.37 ± 0.70	-0.797^b^	r = -0.08	0.426

^a^ Student T test, ^b^Mann-Whitney U test.

Income was coded as <1000 Turkish Lira (TL)=1; 1000–3000 TL = 2; 3000–5000 TL = 3; >5000 TL = 4.

Effect sizes are reported as Cohen’s d for independent-samples t tests, Rosenthal’s standardized effect size (r = |Z|/√N) for Mann–Whitney U tests, and phi (φ) for the chi-square test.

Missing data in the ASD group were limited to the ABC Inappropriate Speech subscale (1/50, 2%), paternal BSI subscales (1/50, 2%), and ADSI developmental scores (3/50, 6%). There were no missing data for the CEBQ subscales or sociodemographic variables used in the ASD–TD group comparisons.

No significant between-group differences were observed across any of the CEBQ subscales. **Table 2** presents the group comparisons. Because maternal age (t = 3.149, p = 0.002) and paternal age (Z = −3.372, p = 0.001) differed significantly between the ASD and TD groups, these variables were initially included as covariates in the MANCOVA. After adjustment for these sociodemographic differences, the multivariate effect of group on CEBQ subscale scores was non-significant (Pillai’s Trace = 0.064, F(8,82) = 0.703, p = 0.688, partial η² = 0.064). Given the marked between-group difference in overall developmental level (t = −11.394, p < 0.001), overall developmental level was additionally included as a covariate in a subsequent model. The multivariate effect of group remained non-significant after this additional adjustment (Pillai’s Trace = 0.116, F(8,81) = 1.332, p = 0.240, partial η² = 0.116), suggesting that the results were robust to adjustment for these baseline differences. Complete-case sensitivity analysis of the MANCOVA additionally adjusted for developmental level yielded a similarly non-significant group effect (Pillai’s Trace = 0.156, F(8,78) = 1.804, p = 0.089).

**Table 2:** Comparison of CEBQ subscale scores between ASD and control groups.

CEBQ subscale	ASD (*n* = 50)	TD (*n* = 43)	*t/z^2^*	Effect size	Effect estimate [95% CI]	*p*
Food responsiveness	8.00 ± 3.49	8.89 ± 3.61	-1.33 ^b^	r = 0.14	−1.00 [−2.00, 0.00]	0.184
Emotional overeating	5.20 ± 1.96	5.42 ± 1.90	-1.06 ^b^	r = 0.11	0.00 [0.00, 0.00]	0.289
Enjoyment of food	15.66 ± 5.58	14.62 ± 5.79	-0.90 ^b^	r = 0.09	1.00 [−1.00, 4.00]	0.371
Desire to drink	8.06 ± 3.48	9.26 ± 4.09	-1.40 ^b^	r = 0.14	−1.00 [−3.00, 0.00]	0.162
Satiety responsiveness	20.77 ± 5.73	21.04 ± 5.45	-0.23 ^a^	d = −0.05	−0.27 [−0.46, 0.36]	0.821
Slowness in eating	8.81 ± 3.53	9.39 ± 3.56	-0.79 ^a^	d = −0.16	−0.58 [−0.57, 0.25]	0.433
Emotional undereating	9.62 ± 4.13	9.87 ± 4.10	-0.25 ^a^	d = −0.06	−0.25 [−0.47, 0.35]	0.773
Food fussiness	7.26 ± 3.14	7.50 ± 3.26	-0.36 ^b^	r = 0.04	0.00 [−2.00, 1.00]	0.721

Values are presented as mean ± standard deviation (SD).

^a^Student T test; effect size reported as Cohen’s d with its 95% confidence interval.

^b^Mann–Whitney U test (reported as standardized z statistic); effect size reported as r (r = |z|/√N) and the Hodges–Lehmann estimate of the location shift with its 95% confidence interval.

The correlations between eating behaviors and age, developmental level, CARS scores, ABC scores and BSI scores were calculated in ASD group. CARS total score and age did not correlate with CEBQ scores. Statistically significant correlations between several CEBQ subscales and these variables were found (**Supplementary Tables S1A–C**). Irritability/agitation/crying was positively correlated with satiety responsiveness (r_s_= 0.336, p = 0.017). Lethargy/social withdrawal showed a negative correlation with enjoyment of food and food fussiness and a positive correlation with satiety responsiveness (r_s_ = −0.317, p = 0.025; r_s_ = −0.325, p = 0.021; r_s_ = 0.312, p = 0.027). Hyperactivity/noncompliance demonstrated positive correlations with satiety responsiveness, slowness in eating and emotional undereating (r_s_= 0.449, p = 0.001; r_s_= 0.399, p = 0.004; r_s_= 0.326, p = 0.021), while exhibiting a negative correlation with food fussiness (r_s_= -0.295, p = 0.037). Fine motor skills were positively correlated with food responsiveness, enjoyment of food (r_s_= 0.296, p = 0.037; r_s_= 0.331, p = 0.019), and desire to drink (r_s_= 0.398, p = 0.004), but negatively correlated with slowness in eating (r= -0.321, p = 0.023). Regarding parental factors, maternal and paternal depressive symptom scores were positively associated with emotional overeating (r_s_= 0.348, p = 0.013; r_s_= 0.321, p = 0.023). Furthermore, fathers’ depressive and somatization symptom scores were each positively correlated with slowness in eating (r_s_= 0.305, p = 0.032; r_s_= 0.322, p = 0.027). After Benjamini–Hochberg false discovery rate correction, only the correlation between satiety responsiveness and ABC-H remained statistically significant (FDR-adjusted p = 0.022) (**Table 3**). Complete-case correlation results were generally comparable to the primary analysis; however, the association between hyperactivity/noncompliance and satiety responsiveness no longer remained significant after FDR correction (adjusted p = 0.066).

**Table 3:** Significant correlations between CEBQ subscales and clinical variables in the ASD group.

CEBQ subscale	Variable	Correlation coefficient	Raw p	BH-adjusted p
Food responsiveness	ADSI-FM	r_s_ =0.296	0.037	0.814
Emotional overeating	BSI-D-F	r_s_ =0.321	0.023	0.222
	BSI-D-M	r_s_ =0.348	0.013	0.222
Enjoyment of food	ABC-L	r_s_ =−0.317	0.025	0.275
	ADSI-FM	r_s_ =0.331	0.019	0.275
Desire to drink	ADSI-FM	r_s_ =0.398	0.004	0.088
Satiety responsiveness	ABC-I	r_s_ =0.336	0.017	0.187
	ABC-L	r_s_ =0.312	0.027	0.198
	ABC-H	r_s_ =0.449	0.001	**0.022**
Slowness in eating	ABC-H	r_s_ =0.399	0.004	0.088
	ADSI-FM	r=−0.321	0.023	0.176
	BSI-D-F	r_s_ =0.305	0.032	0.176
	BSI-S-F	r_s_ =0.322	0.027	0.176
Emotional undereating	ABC-H	r_s_ =0.326	0.021	0.462
Food fussiness	ABC-L	r_s_ =−0.325	0.021	0.407
	ABC-H	r_s_ =−0.295	0.037	0.407

ABC, Aberrant Behavior Checklist; ABC-I, Irritability/Agitation/Crying; ABC-L, Lethargy/Social Withdrawal; ABC-H, Hyperactivity/Noncompliance.

ADSI, Ankara Developmental Screening Inventory; ADSI-FM, Fine Motor Abilities Subscale

BSI, Brief Symptom Inventory; BSI-D-M/F, Depression (Mother/Father); BSI-S-F, Somatization Father

r_s_, Spearman’s rank correlation coefficient; r, Pearson’s correlation coefficient

Only correlations with nominal significance (raw p < 0.05) are presented. Benjamini–Hochberg false discovery rate (FDR) correction was applied separately within each CEBQ subscale; correlations involving each CEBQ subscale constituted a distinct family of tests.

Bold indicates statistical significance after FDR correction.

Separate multivariable linear regression analyses were performed for each CEBQ subscale. The models for food responsiveness, emotional overeating, enjoyment of food, desire to drink, satiety responsiveness, and slowness in eating were statistically significant. Fine motor skills emerged as a nominally significant positive predictor of food responsiveness (β = 0.291, p = 0.041) and desire to drink (β = 0.404, p = 0.012), and as a nominally significant negative predictor of slowness in eating (β = −0.289, p = 0.035). Cognitive–language developmental quotient was a nominally significant negative predictor of emotional overeating (β = −0.333, p = 0.029) and a positive predictor of satiety responsiveness (β = 0.299, p = 0.043) (**Table 4**). Complete regression results, including unstandardized coefficients (B), 95% confidence intervals (CIs), standardized coefficients (β), and p values for all variables included in each model, are provided in **Supplementary Table S2**. However, none of these individual predictor associations remained statistically significant after Benjamini–Hochberg FDR correction across predictor coefficients from the eight regression models (all q ≥ 0.292). The complete-case regression analyses yielded generally comparable findings to the primary analyses, although some differences in nominal statistical significance were observed (**Supplementary Table S3**). Consistent with the primary analyses, none of the predictor-specific associations remained statistically significant after Benjamini–Hochberg false discovery rate correction.

**Table 4:** Summary of multivariable linear regression analyses for CEBQ subscale scores.

CEBQ subscale	Model statistics (R², Adj R², F, p)	Nominally significant association(s)
Food responsiveness	0.084, 0.065, F (1,48)=4.43, *p* = 0.041	**ADSI-FM (β=0.291, *p* = 0.041)**
Emotional overeating	0.226, 0.138, F(5,44)=2.575, *p* = 0.040	**ADSI-CL (β=−0.333, *p* = 0.029)**
Enjoyment of food	0.236, 0.149, F(5,44)=2.722, *p* = 0.032	None
Desire to drink	0.211, 0.141, F(4,45)=3.012, *p* = 0.028	**ADSI-FM (β=0.404, p=0.012)**
Satiety responsiveness	0.266, 0.182, F(5,44)=3.183, *p* = 0.015	**ADSI-CL (β=0.299, *p* = 0.043)**
Slowness in eating	0.217, 0.147, F(4,45)=3.109, *p* = 0.024	**ADSI-FM (β=−0.289, *p* = 0.035)**
Emotional undereating	0.090, −0.013, F(5,44)=0.872, *p* = 0.508	None
Food fussiness	0.162, 0.067, F(5,44)=1.705, *p* = 0.153	None

Adj R², adjusted coefficient of determination; CEBQ, Children’s Eating Behavior Questionnaire; ADSI, Ankara Developmental Screening Inventory; ADSI-FM, Fine Motor Abilities Subscale; ADSI-CL, Cognitive–Language Subscale.

Values shown are the overall model statistics and nominally significant associations (uncorrected p <no><</no> 0.05) identified in each multivariable linear regression model. Standardized regression coefficients (β) are presented for these associations. None of these associations remained statistically significant after false discovery rate (FDR) correction. Complete regression coefficients (B), 95% confidence intervals (CI), standardized regression coefficients (β), and p values for all variables are provided in **Supplementary Table S2**.

Bold indicates statistical significance after FDR correction.

A conceptual summary of the primary between-group findings and the key within-group associations identified after FDR-corrected correlation and multivariable regression analyses is presented in **Figure 1**.

**Figure 1:**
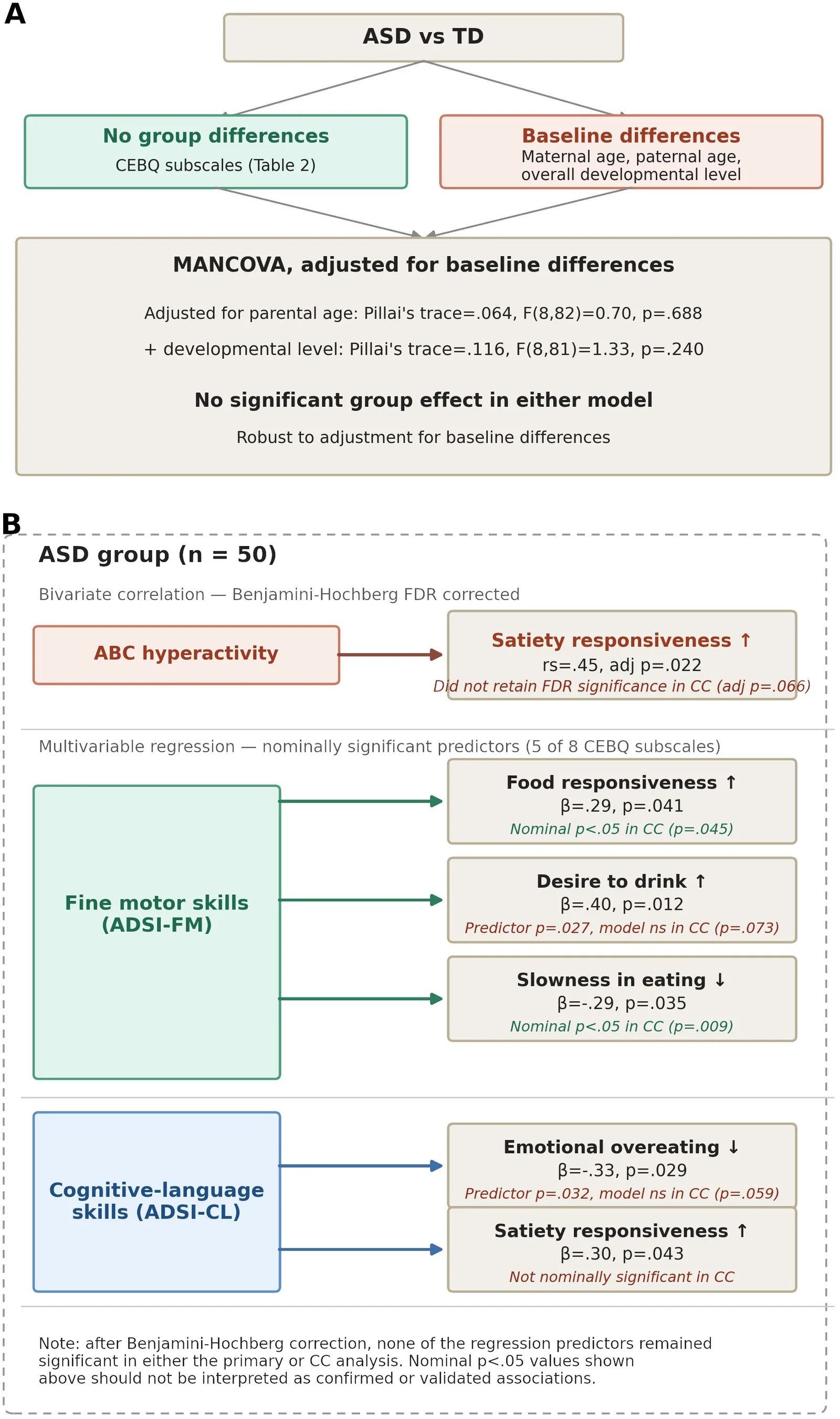
Summary of the main study findings. **(A)** Comparison of CEBQ subscale scores between children with autism spectrum disorder (ASD) and typically developing (TD) children, including multivariate analyses adjusted for baseline differences. **(B)** Within-group associations in children with ASD, showing the FDR-corrected bivariate correlation and nominally significant associations identified in multivariable regression analyses. None of the regression associations remained statistically significant after Benjamini–Hochberg FDR correction. Complete-case sensitivity findings are also indicated. ASD, autism spectrum disorder; TD, typically developing; CEBQ, Children’s Eating Behavior Questionnaire; ABC, Aberrant Behavior Checklist; ADSI-FM, Ankara Developmental Screening Inventory–Fine Motor Abilities; ADSI-CL, Ankara Developmental Screening Inventory–Cognitive–Language; FDR, false discovery rate; CC, complete-case.

## 4 Discussion

Contrary to our initial hypothesis, this case-control study found no significant differences in parent-reported eating behaviors assessed by the CEBQ between preschool-aged children with ASD compared to their TD peers. Although unexpected, this result is consistent with the complex and mixed findings reported in the existing literature. Emond et al. (17) reported that children with ASD exhibited delays in the introduction of solid foods, food selectivity, and feeding difficulties from early infancy. Johnson et al. (18) and Provost et al. (19) reported that children with ASD exhibit more problematic eating and mealtime behaviors compared to their TD peers, and Yeung et al. (14) specifically noted higher levels of food refusal and selectivity in this group. In line with our findings, some previous studies have also reported either non-significant group differences or mixed results regarding eating behaviors in children with ASD. Kral et al. (20) found significant differences only in the food fussiness subscale. Similarly, Hekimci et al. (49) reported that the only domain showing a significant group difference was emotional overeating, which was higher in children with ASD. Öz and Bayhan (21) observed higher rates of food refusal among children with ASD, whereas emotional under-eating was more common among TD children. In contrast, Shmaya et al. (22) reported no significant group differences in food refusal but noted a greater overall prevalence of eating problems in the ASD group. On the other hand, Furthermore, Diolordi et al. (23) did not identify any significant differences in eating problems between children with ASD and their TD peers of preschool age.

Our findings can be interpreted in several ways. Numerous studies have identified selective eating as the most prevalent eating problem among children with ASD (50). However, some researchers have emphasized that early childhood represents a developmental phase during which food selectivity reaches its peak in many children, irrespective of diagnostic status. Although such behaviors are generally transient among typically developing children, they may persist in those with ASD (51–53). This developmental divergence may become more evident with increasing age, as eating difficulties in TD children often resolve spontaneously, whereas autism-related characteristics, such as sensory sensitivities and insistence on sameness, may contribute to the persistence of feeding problems in children with ASD (54, 55). Given that our sample consisted exclusively of preschool-aged children, it is plausible that elevated levels of food selectivity were present in both groups due to age-related developmental factors. This overlap in behavioral characteristics may partially explain the lack of significant group differences observed in our study. Accordingly, the developmental trajectories of eating behaviors in the two groups may not yet have diverged sufficiently for these differences to be detected. Our interpretation is supported by Diolordi et al. (23), who reported no significant group differences in eating problems between children with ASD and their TD peers aged 2 to 5.9 years, whereas a notable difference emerged in the older age group (6 to 9.5 years). Taken together, these findings suggest that developmental stage should be considered when interpreting eating behavior findings across studies.

Another explanation for the absence of significant group differences may relate to our sample selection. Chronic health conditions have been implicated in the etiology of feeding difficulties in autism and are reported to be more prevalent in children with ASD compared to the general population (56). In our study, children with chronic diseases or those taking regular medication were excluded to control for potential confounding variables. However, this exclusion criterion may have resulted in the underrepresentation of children with more severe or complex feeding problems within the ASD group. This issue may be particularly relevant given the marked clinical heterogeneity of ASD, as eating behaviors may vary according to individual clinical characteristics rather than diagnosis alone. Indeed, although Balakireva et al. (57) reported distinct feeding profiles across clinical ASD variants, Matson et al. (58) found no significant differences in feeding problems between children with autistic disorder and PDD-NOS, highlighting the inconsistent findings regarding feeding behaviors across ASD subgroups. Moreover, because our ASD sample was analyzed as a single diagnostic group, potential differences across clinical subgroups could not be explored. Therefore, the characteristics of our ASD sample, including the possible underrepresentation of children with more complex clinical presentations, should be considered when interpreting these findings.

Beyond sample characteristics, cultural context may also help explain the absence of significant differences in CEBQ subscale scores between children with ASD and TD peers. Previous studies have shown that children’s eating behaviors and parental feeding practices vary across cultures and are shaped by family food environments, parental beliefs, feeding practices, and broader sociocultural factors (59–61). Furthermore, parent-reported measures may also be influenced by cultural norms regarding what is considered typical or problematic child behavior (62), an important consideration given that the CEBQ is a parent-report instrument. In the Turkish context, Tekin et al. (63) reported that authoritative and overprotective parenting styles were the most common among parents of children with ASD, with overprotective parenting being positively associated with enjoyment of food. The authors suggested that Turkish parents’ tendency to perceive higher body weight as a sign of health and beauty, together with more controlling feeding practices, may influence children’s eating behaviors. Likewise, Asil et al. (64) reported that food selectivity and food refusal are common but often underestimated in Turkish children with ASD. Consistent with our findings, Hekimci et al. (49) reported significant group differences only for the emotional overeating subscale of the CEBQ, while all other subscales were comparable between children with ASD and TD children. The authors noted that parents commonly continued feeding their children in both groups, a culturally specific pattern that may limit generalizability. Collectively, these findings suggest that cultural differences in parental feeding practices and parent perceptions may contribute to variability in parent-reported eating behaviors across studies.

Finally, the relatively modest sample size may have limited the statistical power to detect subtle between-group differences. Therefore, the possibility that small but potentially meaningful differences remained undetected cannot be excluded.

Contrary to our second hypothesis, autism symptom severity was not associated with eating behaviors in our sample. Although autism severity has frequently been examined as a potential contributor to feeding difficulties in children with ASD, findings across studies remain mixed. Some research reports that eating problems increase with greater autism severity; for example, Crasta et al. (65) identified a significant positive correlation between BAMBI scores and total CARS scores. In contrast, Schreck and Williams (66) found no meaningful effect of ASD severity on eating behaviors. Furthermore, some studies suggest that specific autism-related features such as sensory sensitivities or resistance to change may be more strongly linked to eating problems than overall ASD severity. For example, Martins et al. (67) reported that difficulties with adapting to change and sensory issues were associated with eating problems, even though total CARS scores were not. Taken together, these findings suggest that overall autism severity alone may not adequately explain eating behaviors in children with ASD and that other symptom dimensions may also contribute. Future research should incorporate detailed assessments of sensory processing and behavioral rigidity to better understand the mechanisms underlying eating problems in this population.

Our hypothesis that developmental characteristics would be associated with eating behaviors in the ASD group received only limited support. We found no significant relationship with age, a result consistent with the mixed findings in the literature (37, 53, 68) and potentially constrained by our sample’s narrow age range. Fine motor skills showed nominal positive associations with food responsiveness and desire to drink and a nominal negative association with slowness in eating; however, none of these associations remained statistically significant after FDR correction. Fine motor abilities are relevant to self-feeding, a developmental milestone that relies on fine motor proficiency (69), and previous studies have also shown that children with developmental disorders frequently experience difficulties related to self-feeding and utensil handling (70, 71). Notably, Ooka et al. (71) further highlighted the importance of considering self-feeding function when evaluating feeding difficulties in children with ASD. However, given that the associations observed in the present study did not survive FDR correction, they should be regarded as exploratory and hypothesis-generating and require confirmation in larger studies.

Cognitive-language development showed nominal associations with lower emotional overeating and greater satiety responsiveness, neither of which remained significant after FDR correction. Previous studies examining the relationship between developmental functioning and feeding behaviors in children with ASD have yielded inconsistent findings. While Dominick et al. (72) found no association between cognitive or language abilities and atypical feeding behaviors, Postorino et al. (73) reported lower nonverbal IQ among children with food selectivity. These differences may reflect the distinct eating-related constructs examined across studies. Previous studies have suggested that cognitive and language abilities may be relevant to children’s communication of internal states such as hunger and fullness and to caregivers’ recognition of satiety-related behaviors (54, 74). However, given that these associations did not survive FDR correction, together with the parent-reported assessment of eating behaviors and the cross-sectional design, this interpretation should be considered with caution.

Our hypothesis that behavioral characteristics would be associated with eating behaviors received limited support. Specifically, higher levels of hyperactivity/noncompliance were associated with greater satiety responsiveness. Although previous studies have reported positive associations between hyperactivity and food-approach behaviors, particularly emotional overeating (75, 76), our findings suggest a different pattern in preschool children with ASD. One possible explanation is that children with higher levels of hyperactivity may be more easily distracted during meals, leading caregivers to perceive them as becoming full more quickly or disengaging from eating. This interpretation is consistent with Fuemmeler et al. (75), who proposed that children with ADHD symptoms are more susceptible to environmental distractions during meals, which may result in reduced interest in eating or premature termination of meals. Although this mechanism remains speculative, it may partly explain the positive association between hyperactivity/noncompliance and satiety responsiveness observed in our sample. However, this finding should be interpreted cautiously, as the association did not remain significant after FDR correction in the complete-case sensitivity analysis.

The final component of our second hypothesis anticipated an association between parental mental health and children’s eating behaviors. Contrary to our hypothesis, we did not identify significant independent associations between parental mental health and children’s eating behaviors. However, this finding should not be interpreted as indicating that parental psychological well-being is unimportant in the context of feeding difficulties. Previous studies have consistently shown that feeding problems in children with ASD are associated with substantial parental distress and psychological burden (34, 77). Moreover, mealtimes in families of children with ASD are frequently characterized by heightened stress and anxiety rather than positive family interactions (78). Rather than exerting a direct effect on children’s eating behaviors, parental psychological symptoms may influence feeding through more proximal mechanisms. For example, Zlomke et al. (35) reported that although maternal anxiety was not directly associated with children’s feeding problems, it was related to more maladaptive mealtime emotions and feeding strategies, which were stronger predictors of feeding difficulties. Similarly, Rouphael et al. (79) emphasized that parental distress during mealtimes may adversely affect adherence to feeding interventions. Because our study assessed only general symptoms of parental depression and anxiety and did not evaluate mealtime-specific emotions, feeding practices, or parent–child mealtime interactions, these more proximal processes may have remained undetected. Future studies incorporating these factors may provide a more comprehensive understanding of how parental mental health influences eating behaviors in children with ASD.

Our study has several limitations. First, the small sample size restricts the generalizability of the findings, and studies with larger samples may yield more robust results. Although the *post hoc* sensitivity analysis indicated adequate power to detect medium-to-large between-group differences, smaller but potentially clinically meaningful differences may have remained undetected. Therefore, the absence of significant between-group differences should be interpreted with caution. Second, the assessments of behavioral problems, eating behaviors, and parental mental health were all based on parent-report questionnaires. While this method is commonly used, it may be subject to reporting bias. For instance, although our study relied on parental reports to assess eating behaviors, Aponte and Romanczyk (80) used both the BAMBI and direct mealtime observations in their study and found no significant association between BAMBI scores and observed behaviors, except for negative vocalizations. Future studies may benefit from incorporating multi-method assessment approaches, including observational measures, to improve the accuracy and ecological validity of eating behavior evaluations in children with ASD. In addition, excluding parents who were receiving psychiatric treatment may have reduced the representation of families with more severe parental psychopathology, potentially attenuating the observed associations and limiting the generalizability of our findings. Another limitation is the use of the ADSI to assess developmental levels in children with ASD, as this instrument lacks established validity and reliability for ASD populations. Furthermore, due to the cross-sectional design of the study, causal relationships cannot be established, which limits the ability to determine the directionality of the observed associations. Although multiple comparisons were addressed using the Benjamini–Hochberg false discovery rate correction and multivariable regression analyses, the within-group findings require confirmation in larger longitudinal studies. Future research employing longitudinal designs and standardized developmental assessment tools validated for ASD populations may offer more robust insights into the mechanisms underlying eating behaviors in this group. Despite these limitations, our study has notable strengths. It focuses on preschool period, a critical period for the development of eating behaviors, and includes an evaluation of parental mental health, thereby offering a more comprehensive view of the factors that may influence eating behaviors in children with ASD. These aspects contribute meaningful insights to the literature and underscore the need for continued research in this area.

## 5 Conclusion

The present findings indicate that preschool children with ASD did not differ from their TD peers in parent-reported eating behaviors. Within the ASD group, the observed patterns suggest that developmental and behavioral characteristics may contribute to individual variability in specific eating behavior dimensions. However, the limited statistical robustness of these associations indicates that they should not yet be considered established correlates of eating behavior in ASD. Rather, they may represent candidate factors warranting further investigation. Given the cross-sectional design and relatively small sample, larger longitudinal studies are needed to determine whether these patterns can be replicated and to clarify their potential clinical relevance.

## Data Availability

The raw data supporting the conclusions of this article will be made available by the authors, without undue reservation.
