# Supplementary material for: Eating behaviors in preschoolers with autism spectrum disorders: associations with developmental and behavioral features and parental mental health
Source: Front Psychiatry. 2026 Oct 15;17:1902816. doi: 10.3389/fpsyt.2026.1902816 (PMC13735782; doi:10.3389/fpsyt.2026.1902816)
Supplement: Supplementary file 1 [file Table1.docx]

| **Table S1A Correlations Between CEBQ Subscale Scores and Age, Aberrant Behavior Checklist (ABC) Scores, and Childhood Autism Rating Scale (CARS)** | | | | | | | |
| --- | --- | --- | --- | --- | --- | --- | --- |
| **CEBQ Subscale** | **Age** | **ABC-I** | **ABC-L** | **ABC-S** | **ABC-H** | **ABC-IS** | **CARS** |
| Food responsiveness | rₛ=0.031  p=0.832 | rₛ=-0.093  p=0.522 | rₛ=-0.162  p=0.261 | rₛ=0.038  p=0.795 | rₛ=0.060  p=0.680 | rₛ=-0.060  p=0.680 | rₛ=-0.071  p=0.626 |
| Emotional overeating | rₛ=−0.034  p=0.812 | rₛ=0.211  p=0.141 | rₛ=0.218  p=0.128 | rₛ=0.223  p=0.120 | rₛ=0.093  p=0.522 | rₛ=-0.267  p=0.061 | rₛ=0.223  p=0.120 |
| Enjoyment of food | rₛ=−0.141  p=0.330 | rₛ=-0.183  p=0.203 | **rₛ=-0.317**  **p=0.025** | rₛ=-0.140  p=0.333 | rₛ=-0.245  p=0.087 | rₛ=-0.016  p=0.912 | rₛ=-0.238  p=0.096 |
| Desire to drink | rₛ=0.178  p=0.215 | rₛ=0.084  p=0.561 | rₛ=-0.097  p=0.503 | rₛ=0.163  p=0.259 | rₛ=-0.031  p=0.833 | rₛ=-0.181  p=0.209 | rₛ=0.086  p=0.551 |
| Satiety responsiveness | r=−0.014  p=0.921 | **rₛ=0.336**  **p=0.017** | **rₛ=0.312**  **p=0.027** | rₛ=0.184  p=0.201 | **rₛ=0.449**  **p=0.001** | rₛ=0.078  p=0.590 | rₛ=0.190  p=0.186 |
| Slowness in eating | r=0.179  p=0.215 | rₛ=0.218  p=0.128 | rₛ=0.258  p=0.071 | rₛ=0.226  p=0.114 | **rₛ=0.399**  **p=0.004** | rₛ=0.141  p=0.328 | rₛ=0.114  p=0.431 |
| Emotional undereating | r=−0.099  p=0.494 | rₛ=0.218  p=0.128 | rₛ=0.069  p=0.635 | rₛ=0.101  p=0.485 | **rₛ=0.326**  **p=0.021** | rₛ=0.114  p=0.429 | rₛ=0.117  p=0.420 |
| Food fussiness | rₛ=−0.002  p=0.991 | rₛ=-0.230  p=0.107 | **rₛ=-0.325**  **p=0.021** | rₛ=-0.013  p=0.927 | **rₛ=-0.295**  **p=0.037** | rₛ=0.022  p=0.882 | rₛ=-0.100  p=0.489 |
| ABC: Aberrant Behavior Checklist; ABC-I: Irritability/Agitation/ Crying; ABC-L: Lethargy/Social Withdrawal; ABC-S: Stereotypic Behavior; ABC-H: Hyperactivity/Noncompliance; ABC-IS: Inappropriate Speech. CARS: Childhood Autism Rating Scale.  r = Pearson's correlation coefficient; rₛ = Spearman's rank correlation coefficient.  Statistically significant correlations (p < 0.05) are shown in bold. | | | | | | | |

| **Table S1B Correlations Between CEBQ Subscale Scores and Ankara Developmental Screening Inventory (ADSI) Scores** | | | | | |
| --- | --- | --- | --- | --- | --- |
| **CEBQ Subscale** | **ADSI-GDS** | **ADSI-CL** | **ADSI-FM** | **ADSI-GM** | **ADSI-SS** |
| Food responsiveness | rₛ=-0.020  p=0.893 | rₛ=-0.008  p=0.959 | **rₛ=0.296**  **p=0.037** | rₛ=-0.026  p=0.856 | rₛ=-0.017  p=0.907 |
| Emotional overeating | rₛ=-0.214  p=0.136 | rₛ=-0.266  p=0.062 | rₛ=0.033  p=0.819 | rₛ=0.068  p=0.641 | rₛ=-0. 232  p=0. 105 |
| Enjoyment of food | rₛ=0.110  p=0.447 | rₛ=0.004  p=0.978 | **rₛ=0.331**  **p=0.019** | rₛ=0.053  p=0.717 | rₛ=0.222  p=0.122 |
| Desire to drink | rₛ=0.197  p=0.171 | rₛ=0.093  p=0.523 | **rₛ=0.398**  **p=0.004** | rₛ=-0.073  p=0.614 | rₛ=0.168  p=0.243 |
| Satiety responsiveness | r=-0.143  p=0.321 | r=-0.190  p=0.185 | r=-0.151  p=0.296 | r=-0.173  p=0.229 | rₛ=-0.037  p=0.799 |
| Slowness in eating | r=-0.003  p=0.986 | r=-0.043  p=0.768 | **r=-0.321**  **p=0.023** | r=0.098  p=0.500 | rₛ=-0.162  p=0.260 |
| Emotional undereating | r=-0.011  p=0.939 | r=0.020  p=0.889 | r=-0.052  p=0.721 | r=0.161  p=0.263 | rₛ=-0.102  p=0.482 |
| Food fussiness | rₛ=0.196  p=0.173 | rₛ=0.082  p=0.572 | rₛ=0.243  p=0.089 | rₛ=0.019  p=0.896 | rₛ=0.109  p=0.449 |
| ADSI: Ankara Developmental Screening Inventory; ADSI-GDS: General Developmental Score; ADSI-CL: Cognitive–Language Subscale; ADSI-FM: Fine Motor Abilities Subscale; ADSI-GM: Gross Motor Abilities Subscale; ADSI-SS: Social Skills and Self-Care Subscale.  r = Pearson's correlation coefficient; rₛ = Spearman's rank correlation coefficient.  Statistically significant correlations (p < 0.05) are shown in bold. | | | | | |

| **Table S1C Correlations Between CEBQ Subscale Scores and Maternal and Paternal Brief Symptom Inventory (BSI) Scores** | | | | | | | | | | |
| --- | --- | --- | --- | --- | --- | --- | --- | --- | --- | --- |
| **CEBQ Subscale** | **BSI-A-F** | **BSI-D-F** | **BSI-NS-F** | **BSI-**  **S-F** | **BSI-H-F** | **BSI-A-M** | **BSI-D-M** | **BSI-NS-M** | **BSI-**  **S-M** | **BSI-H-M** |
| Food responsiveness | r=0.075  p=0.607 | r=0.085  p=0.558 | r=0.049  p=0.736 | r=0.085  p=0.559 | r=0.073  p=0.612 | r=0.057  p=0.697 | r=0.131  p=0.366 | r=0.100  p=0.491 | r=0.151  p=0.294 | r=0.062  p=0.666 |
| Emotional overeating | r=0.251  p=0.078 | **r=0.321**  **p=0.023** | r=0.259  p=0.070 | r=0.173  p=0.229 | r=0.097  p=0.501 | r=0.249  p=0.082 | **r=0.348**  **p=0.013** | r=0.243  p=0.090 | r=0.181  p=0.208 | r=0.156  p=0.281 |
| Enjoyment of food | r=-0.197  p=0.170 | r=-0.251  p=0.079 | r=-0.171  p=0.235 | r=-0.068  p=0.640 | r=-0.001  p=0.993 | r=-0.161  p=0.263 | r=-0.130  p=0.368 | r=-0.099  p=0.492 | r=-0.064  p=0.661 | r=-0.104  p=0.474 |
| Desire to drink | r=-0.011  p=0.942 | r=0.194  p=0.178 | r=0.002  p=0.991 | r=0.197  p=0.171 | r=0.056  p=0.701 | r=-0.040  p=0.783 | r=0.021  p=0.884 | r=-0.074  p=0.611 | r=0.098  p=0.498 | r=-0.048  p=0.739 |
| Satiety responsiveness | r=0.246  p=0.085 | r=0.256  p=0.073 | r=0.171  p=0.234 | r=0.188  p=0.191 | r=0.159  p=0.271 | r=0.184  p=0.200 | r=0.144  p=0.320 | r=0.078  p=0.589 | r=0.128  p=0.376 | r=0.170  p=0.237 |
| Slowness in eating | r=0.200  p=0.165 | **r=0.305**  **p=0.032** | r=0.176  p=0.222 | **r=0.322**  **p=0.027** | r=0.172  p=0.231 | r=0.095  p=0.511 | r=0.119  p=0.411 | r=0.060  p=0.680 | r=0.129  p=0.371 | r=0.071  p=0.624 |
| Emotional undereating | r=0.184  p=0.200 | r=0.162  p=0.261 | r=0.098  p=0.500 | r=0.145  p=0.316 | r=0.108  p=0.454 | r=0.222  p=0.121 | r=0.162  p=0.260 | r=0.185  p=0.198 | r=0.197  p=0.169 | r=0.163  p=0.259 |
| Food fussiness | r=-0.149  p=0.302 | r=-0.031  p=0.833 | r=-0.040  p=0.781 | r=0.025  p=0.865 | r=0.049  p=0.734 | r=-0.193  p=0.179 | r=-0.174  p=0.228 | r=-0.078  p=0.590 | r=-0.101  p=0.485 | r=-0.083  p=0.568 |
| BSI: Brief Symptom Inventory; BSI-A-M/F: Anxiety (Mother/Father); BSI-D-M/F: Depression (Mother/Father); BSI-S-M/F: Somatization (Mother/Father); BSI-NS-M/F: Negative Self (Mother/Father); BSI-H-M/F: Hostility (Father).  All correlation coefficients are Spearman's rank correlation coefficients.  Statistically significant correlations (p < 0.05) are shown in bold. | | | | | | | | | | |

| **Supplementary Table S2 Multiple linear regression analyses examining factors associated with Children's Eating Behaviour Questionnaire (CEBQ) subscale scores** | | | | | | | | | | | | | | | | |
| --- | --- | --- | --- | --- | --- | --- | --- | --- | --- | --- | --- | --- | --- | --- | --- | --- |
|  | **Food responsiveness** |  | **Emotional overeating** |  | **Enjoyment of food** |  | **Desire to drink** |  | **Satiety responsiveness** |  | **Slowness in eating** |  | **Emotional undereating** |  | **Food fussiness** |  |
| **Predictor** | **B (CI)**  **β** | **p** | **B (CI)**  **β** | **p** | **B (CI)**  **β** | **p** | **B (CI)**  **β** | **p** | **B (CI)**  **β** | **p** | **B (CI)**  **β** | **p** | **B (CI)**  **β** | **p** | **B (CI)**  **β** | **p** |
| **ADSI-FM** | 0.304 (0.013–0.594)  0.291 | **0.041** |  |  | 0.272 (−0.053-0.597)  0.231 | 0.099 | 0.528 (0.121-0.936) 0.404 | **0.012** |  |  | −6.519 (−12.573-−0.465)  −0.289 | **0.035** |  |  | 0.197 (−0.151-0.546) 0.161 | 0.260 |
| **Log ABC-I** |  |  | 0.033 (−0.093-0.159)  0.081 | 0.597 |  |  |  |  | 3.417 (−2.489-9.322) 0.200 | 0.250 | −0.596 (−4.271-3.079)  −0.057 | 0.745 | 0.991 (−3.954-5.937)  0.081 | 0.688 | 0.007 (−0.218-0.232) 0.012 | 0.950 |
| **ADSI-CL** |  |  | −0.330 (−0.624-−0.036)  −0.333 | **0.029** |  |  |  |  | 12.341 (0.409-24.273) 0.299 | **0.043** |  |  |  |  |  |  |
| **Log CARS** |  |  | −0.078 (−0.424-0.269)  −0.068 | 0.653 | −0.155 (−0.600-0.289) −0.102 | 0.485 |  |  | 8.721 (−5.387-22.829) 0.184 | 0.219 |  |  |  |  |  |  |
| **Log BSI-D-F** |  |  | 0.071 (−0.032-0.173)  0.223 | 0.171 | −0.079 (−0.202-0.045) −0.184 | 0.206 | 0.039 (−0.128-0.206) 0.083 | 0.639 | 0.798 (−3.080-4.676) 0.060 | 0.680 | 1.725 (−0.641-4.091)  0.211 | 0.149 |  |  |  |  |
| **Log BSI-D-M** |  |  | 0.059 (−0.047-0.164)  0.178 | 0.268 |  |  |  |  |  |  |  |  |  |  |  |  |
| **Log ABC-L** |  |  |  |  | −0.134 (−0.293-0.025) −0.302 | 0.097 |  |  |  |  |  |  |  |  | −0.154 (−0.333-0.025) −0.335 | 0.090 |
| **Log ABC-H** |  |  |  |  | 0.044 (−0.146-0.234)  0.085 | 0.643 |  |  | 3.398 (−2.168-8.964) 0.212 | 0.225 | 2.319 (−1.178-5.817)  0.234 | 0.188 | 2.518 (−1.970-7.005) 0.217 | 0.264 | 0.061 (−0.165-0.286) 0.113 | 0.590 |
| **ADSI-GDS** |  |  |  |  |  |  | −0.080 (−0.693-0.533) −0.041 | 0.794 |  |  |  |  |  |  |  |  |
| **Log BSI-S-F** |  |  |  |  |  |  | 0.053 (−0.039-0.145) 0.203 | 0.250 |  |  |  |  |  |  |  |  |
| **Log BSI-A-M** |  |  |  |  |  |  |  |  |  |  |  |  | −0.178 (−2.345-1.990) −0.042 | 0.869 | −0.035 (−0.100-0.030) −0.176 | 0.289 |
| **Log BSI-S-M** |  |  |  |  |  |  |  |  |  |  |  |  | 0.308 (−1.794-2.411) 0.064 | 0.769 |  |  |
| **Log BSI-NS-M** |  |  |  |  |  |  |  |  |  |  |  |  | 0.152 (−1.791-2.094) 0.036 | 0.876 |  |  |
| **Model**  **Summary** |  |  |  |  |  |  |  |  |  |  |  |  |  |  |  |  |
| **R²** | 0.084 |  | 0.226 |  | 0.236 |  | 0.211 |  | 0.266 |  | 0.217 |  | 0.090 |  | 0.162 |  |
| **Adjusted R²** | 0.065 |  | 0.138 |  | 0.149 |  | 0.141 |  | 0.182 |  | 0.147 |  | −0.013 |  | 0.067 |  |
| **F** | (1,48) = 4.43 |  | (5,44) = 2.575 |  | (5,44) = 2.722 |  | (4,45) = 3.012 |  | (5,44) = 3.183 |  | (4,45) = 3.109 |  | (5,44) = 0.872 |  | (5,44) = 1.705 |  |
| **p** | **0.041** |  | **0.040** |  | **0.032** |  | **0.028** |  | **0.015** |  | **0.024** |  | 0.508 |  | 0.153 |  |
| ABC: Aberrant Behavior Checklist; ABC-I: Irritability/Agitation/ Crying; ABC-L: Lethargy/Social Withdrawal; ABC-H: Hyperactivity/Noncompliance.  CARS: Childhood Autism Rating Scale.  ADSI: Ankara Developmental Screening Inventory; ADSI-GDS: General Developmental Score; ADSI-CL: Cognitive–Language Subscale; ADSI-FM: Fine Motor Abilities Subscale  BSI: Brief Symptom Inventory; BSI-A-M/F: Anxiety (Mother/Father); BSI-D-M/F: Depression (Mother/Father); BSI-S-M/F: Somatization (Mother/Father); BSI-NS-M/F: Negative Self (Mother/Father).  **Notes:** Variables prefixed with Log were logarithmically transformed before regression analyses to improve normality. Values are presented as unstandardized regression coefficient (B) with 95% confidence interval (CI), standardized regression coefficient (β), and p value. R², adjusted R², F statistic, and model p value are presented for each regression model. Statistically significant associations (p < 0.05) are shown in bold. | | | | | | | | | | | | | | | | |

| **Table S3 Comparison of primary and complete-case multivariable linear regression analyses for CEBQ subscale scores** | | | | |
| --- | --- | --- | --- | --- |
| **CEBQ Subscale** | **Analysis** | **N** | **Model statistics**  **(R², Adj R², F, p)** | **Nominally significant independent predictor(s)** |
| **Food responsiveness** | Primary analysis | 50 | 0.084, 0.065, F(1,48)=4.43,  *p*=0.041 | **ADSI-FM** **(β=0.291, *p*=0.041)** |
|  | Complete-case analysis | 47 | 0.086, 0.066, F(1,45)=4.256,  *p*=0.045 | **ADSI-FM (β=0.294, *p*=0.045)** |
| **Emotional overeating** | Primary analysis | 50 | 0.226, 0.138, F(5,44)=2.575,  *p*=0.040 | **ADSI-CL (β=−0.333, *p*=0.029)** |
|  | Complete-case analysis | 46 | 0.226, 0.129, F(5,40)=2.336,  *p*=0.059 | **ADSI-CL (β=−0.349, *p*=0.032)** |
| **Enjoyment of food** | Primary analysis | 50 | 0.236, 0.149, F(5,44)=2.722,  *p*=0.032 | None |
|  | Complete-case analysis | 46 | 0.252, 0.158, F(5,40)=2.692,  *p*=0.035 | None |
| **Desire to drink** | Primary analysis | 50 | 0.211, 0.141, F(4,45)=3.012,  *p*=0.028 | **ADSI-FM (β=0.404, p=0.012)** |
|  | Complete-case analysis | 46 | 0.184, 0.105, F(4,41)=2.317,  *p*=0.073 | **ADSI-FM (β=0.381, *p*=0.027)** |
| **Satiety responsiveness** | Primary analysis | 50 | 0.266, 0.182, F(5,44)=3.183,  *p*=0.015 | **ADSI-CL (β=0.299, *p*=0.043)** |
|  | Complete-case analysis | 46 | 0.251, 0.157, F(5,40)=2.678,  *p*=0.035 | None |
| **Slowness in eating** | Primary analysis | 50 | 0.217, 0.147, F(4,45)=3.109,  *p*=0.024 | **ADSI-FM (β=−0.289, *p*=0.035)** |
|  | Complete-case analysis | 46 | 0.267, 0.196, F(4,41)=3.738,  *p*=0.011 | **ADSI-FM (β=−0.374, *p*=0.009)** |
| **Emotional undereating** | Primary analysis | 50 | 0.090, −0.013, F(5,44)=0.872,  *p*=0.508 | None |
|  | Complete-case analysis | 49 | 0.079, −0.028, F(5,43)=0.739,  *p*=0.598 | None |
| **Food fussiness** | Primary analysis | 50 | 0.162, 0.067, F(5,44)=1.705,  *p*=0.153 | None |
|  | Complete-case analysis | 46 | 0.164, 0.059, F(5,40)=1.565,  *p*=0.192 | None |
| Adj R²: adjusted coefficient of determination; CEBQ: Children's Eating Behaviour Questionnaire; ADSI: Ankara Developmental Screening Inventory; ADSI-FM: Fine Motor Abilities Subscale; ADSI-CL: Cognitive–Language Subscale.  Note: Primary analyses were conducted using the mean-imputed ASD sample (N=50). Complete-case sensitivity analyses were conducted without imputation, with cases containing missing data in variables included in the respective regression model excluded listwise; therefore, sample sizes varied across models.  Complete-case models retained the same predictor sets as the corresponding primary (imputed) regression models, without re-selection of predictors. Values shown are the overall model statistics and independent predictors with nominal p<0.05. Standardized regression coefficients (β) are presented for nominally significant predictors. | | | | |
